# Fast Blue RR—Siloxane Derivatized Materials Indicate Wound Infection Due to a Deep Blue Color Development

**DOI:** 10.3390/ma8105329

**Published:** 2015-09-25

**Authors:** Doris Schiffer, Gregor Tegl, Robert Vielnascher, Hansjoerg Weber, Rainer Schoeftner, Herfried Wiesbauer, Eva Sigl, Andrea Heinzle, Georg M. Guebitz

**Affiliations:** 1 ACIB—Austrian Centre of Industrial Biotechnology, Konrad-Lorenz Strasse 203630 Tuln, Graz 8010, Austria; andrea.heinzle@qualizyme.com (A.H.); guebitz@boku.ac.at (G.M.G.); 2 Department of Environmental Biotechnology, University of Natural Resources and Life Sciences Vienna, Konrad Lorenz Strasse 20, 3430 Tulln an der Donau, Vienna 1180, Austria; gregor.tegl@boku.ac.at (G.T.); robert.vielnascher@boku.ac.at (R.V.); 3 Department of Organic Chemistry, Technical University Graz, Stremayrgasse 16, 8010 Graz, Austria; hansjoerg.weber@tugraz.at; 4 Functional Surfaces & Nanostructures, PROFACTOR GmbH, Im Stadtgut A2, Steyr-Gleink 4407, Austria; rainer.schoeftner@profactor.at (R.S.); herfried.wiesbauer@profactor.at (H.W.); 5 Qualizyme GmbH, Neue Stiftingtalstrasse 2, Graz 8010, Austria; eva.sigl@qualizyme.com

**Keywords:** immobilization, Fast Blue RR, alkoxysilane derivatized Fast Blue RR, myeloperoxidase, infection detection

## Abstract

There is a strong need for simple and fast methods for wound infection determination. Myeloperoxidase, an immune system-derived enzyme was found to be a suitable biomarker for wound infection. Hence, alkoxysilane-derivatized Fast Blue RR was immobilized via simple hydrolytic polymerization. The resulting enzyme-responsive siloxane layers were incubated with myeloperoxidase, wound fluid or hemoglobin. The reaction was monitored via HPLC measurements and the color development quantified spectrophotometrically. Myeloperoxidase was indeed able to oxidize immobilized Fast Blue RR leading to a blue colored product. No conversion was detected in non-infected wound fluids. The visible color changes of these novel materials towards blue enable an easy distinction between infected and non-infected wound fluids.

## 1. Introduction

Standard procedures for wound infection detection are time consuming (microbiology) or show limited reliability due to the judgement of the classical clinical signs, redness (*rubor*), heat (*calor*), swelling (*tumor*), and pain (*dolor*) or due to the detection of signals specific to secondary wounds [1,2,3]. Hence, alternative methods based on the assessment of biomarkers like metabolites, enzymes or microbes have been suggested [4]. New point-of-care devices should facilitate the diagnosis and treatment of chronic wounds [5]. Various enzymes of the immune system accumulate in the wound fluid during an infection, having a potential as biomarkers for infection detection [6]. Myeloperoxidase (MPO), an enzyme derived from the neutrophils is one of the first enzymes present on site of injury. During the oxidative burst, MPO uses the generated hydrogen peroxide to build highly reactive HOCl, and contributes to the destruction of invading bacteria. Myeloperoxidase shows about ten times higher activities in infected wounds compared to non-infected wounds [7]. The potential of this enzyme was furthermore confirmed in a correlation study with silver standard wound diagnostics [7]. New substrates for myeloperoxidase were tested including Fast Blue RR (4-benzoylamino-2,5-dimethoxybenzenediazonium chloride hemi (zinc chloride) salt) that show significant differences in color development and in the indication of infections in wounds [8].

Fast Blue RR salt is commonly used for the detection of esterase and alkaline phosphatase activities in histochemical and colorimetric analysis [9]. In these reactions, naphthyl derivatives are applied as substrates and the enzymatic release of naphthol is followed via a coupling reaction with a diazonium salt such as Fast Blue RR. These reactions are usually performed in basic media and the formations of colored derivatives take only a few minutes. Applications are found in assays for the determination of esterases as pro-drug target in prostate cancer, or to determine the content of alkylresorcinols (ARs) in ground and whole cereal grains [10,11].

Diagnostic devices in wound care should deliver fast results while being user friendly. Hence, simple handling of point-of-care devices is often achieved by immobilization of the diagnosing compound onto carrier materials. Obviously simple immobilization strategies are essential for large scale production of test strips. Alkoxysilane based substrates would allow the formation of functionalized siloxane layers on a large variety of material surfaces via simple hydrolytic polymerization. Such simple enzyme-responsive surfaces could facilitate wound infection diagnosis and treatment.

## 2. Results and Discussion

**Figure 1:**
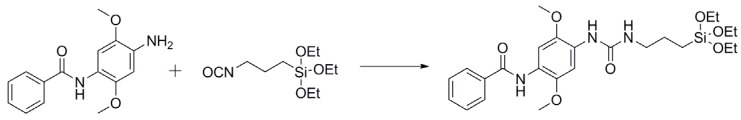
Derivatized Fast Blue RR:Fast blue RR was coupled to 3-(triethoxysilyl)propyl isocyanate. NMR measurements confirm the structure of the stable product.

Recent studies confirmed the suitability of myeloperoxidase (MPO) for infection detection in wounds [8]. Significant differences in enzyme activity were observed comparing infected and non-infected wound fluids, using the well-known substrate guaiacol that can be spectrophotometrically detected. However, it is not possible to covalently immobilize guaiacol without rendering it inactive towards MPO mediated oxidation.. Hence, Fast Blue RR was covalently coupled to alkoxysilanes allowing the simple formation of enzyme-responsive siloxane layers on a variety of surfaces. Figure 1 shows the coupling reaction and the final product that was analyzed with NMR spectroscopy.

**Figure 2:**
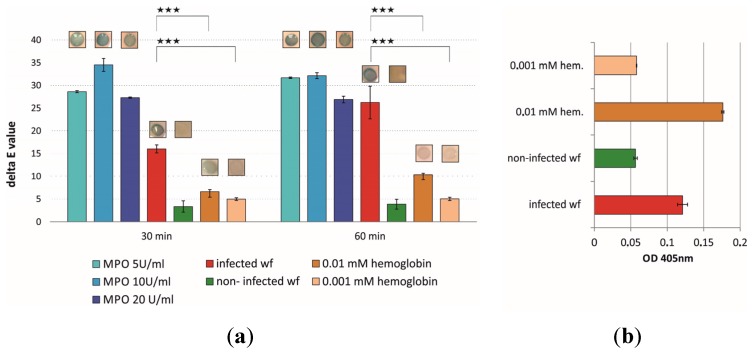
(**a**) Color formation of immobilized alkoxysilane-derivatized Fast Blue RR upon incubation with MPO and wound fluids: alkoxysilane-derivatized Fast Blue RR was polymerized and the resulting enzyme-responsive siloxane layer incubated with different enzyme activities of pure MPO, infected and non-infected wound fluids as well as different concentrations of hemoglobin over a time period of 30 min and 60 min. The blue color formation upon conversion of the substrate was quantified with a ColorLite sph850 spectrophotometer and is given as in delta E values. Two-sample t-tests assuming equal variances were performed, *p*-values equal or less than 0.001 were considered as significant (***). (**b**) The hemoglobin concentration of the infected and non-infected wound fluid was determined prior to the immobilization experiments.

Simple hydrolytic polymerization of this enzyme-responsive substrate enables the application in test strips for early-stage wound infection detection. Thereby it is essential to consider cross reactions with hemoglobin, likewise present in wound fluids. After immobilization of the substrate onto silica plates as model carrier, the reactivity was tested with pure enzyme (MPO), hemoglobin as well as with infected and non-infected wound fluids. A fast color change towards blue (10–30 min) was recorded (Figure 2). The hemoglobin content of the wound fluid was determined prior to the incubation with the substrate and was found to be 0.007 mM. Figure 2 shows the blue color development of the enzyme-responsive siloxane layer upon incubation. A significant difference in the delta E values was observed with infected compared to non-infected wound fluids with a p value of less than 0.001. The color development in Figure 2 shows the progression from 30 to 60 min. An incubation interval of 30 min already provides a statistically significant discrimination between infected and non-infected wound fluids. Cross-reactions with hemoglobin occur, but the delta E values show 60% less color development in samples with a higher hemoglobin content compared to the infected wound fluid sample. Significant differences, independent of the time interval are indicated in Figure 2, representing p values of less than 0.001. Infection detection in wounds still consititutes a challenging topic in medicine. New detection methods of elevated enzyme activity in wounds include ELISA based assays and colorimetric assays. A combination of both, like in the SIEFED technique (Specific Immuno Extraction Followed by Enzymatic Detection) could also play a role in MPO determination in the future [12]. It is moreover important to shorten the reaction times as well as the pre-handling procedures. Enzyme-triggered color changes on a surface in a short time period represent a suitable tool for a fast assumption of the wound status.

### 2.1. LC-ESI-TOF

**Figure 3:**
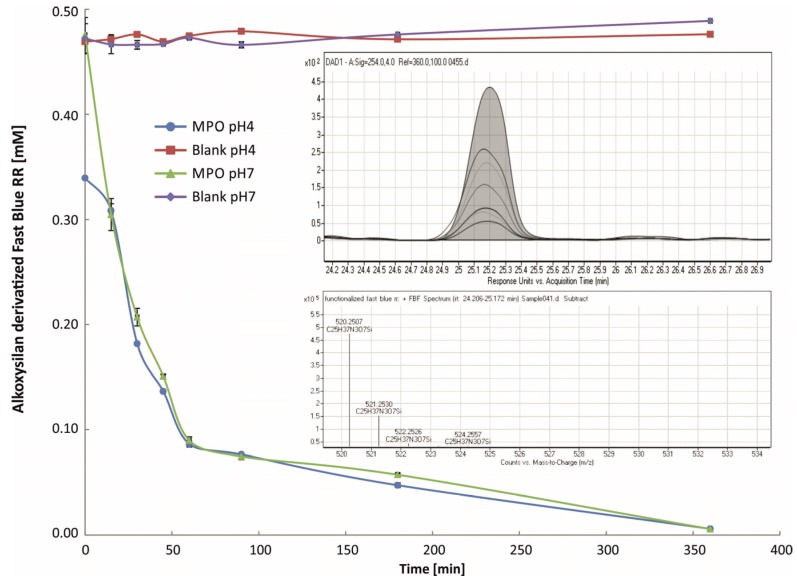
LC-MS TOF analysis of alkoxysilane-derivatized Fast Blue RR conversion by MPO: A substantial conversion of substrate could be observed already after several minutes at both pH values. After 6 h incubation time no substrate could be detected anymore by LC-ESI TOF. The graphs inside the figure depict the decrease of substrate illustrated as the function the substrate mass being 520.25.

In order to investigate MPO oxidation of alkoxysilane-derivatized Fast Blue RR, LC-MS experiments were conducted. Two different pH values were investigated comparing the reaction kinetics at physiological conditions and at acidic pH. Similar to other MPO substrates like guaiacol, LC-ESI-TOF indicated that oxidation of alkoxysilane-derivatized Fast Blue RR led to the formation of a variety of oligomers [13]. Hence, the reaction was monitored based on the consumption of alkoxysilane-derivatized Fast Blue RR. For both pH values a substantial decrease within 60 min was observed by LC and ESI-TOF (Figure 3). An immediate Fast Blue RR consumption of alkoxysilane-derivatized Fast Blue RR was observed at pH 4, however, similar results at both pH values were obtained for the subsequent time measurements. It is supposed the alkoxysilane-derivatized Fast Blue RR is less stable under acidic conditions, which leads to a partial degradation of the substrate prior to enzymatic conversion. The LC data shows a fast consumption of the enzyme responsive material with full conversion observed already after 6 h.

Both the color reaction of the enzyme-responsive siloxane layer and LC-ESI TOF results reveal the suitability of derivatized Fast Blue RR being an effective system for a sensitive detection of elevated MPO activity after short time. The fast response within the first minutes of reaction time renders this detection system a promising candidate for the incorporation into point-of-care diagnostics. Additionally, the functional spacer facilitates the attachment on various surfaces.

## 3. Experimental Section

### 3.1. Wound Fluid Collection and Microbiological Determination

Before sample collection, wounds were cleaned with 0.9% NaCl (Sigma Aldrich, St. Louis, MO, USA) to remove superficial bacteria. Wound fluid was taken with nylon swabs, of the most contaminated and/or deep site of the wound bed and/or wound edges. The samples were analyzed with MALDI TOF techniques, addressing the analyses of the occurring species, and Gram staining-dependent microscopy evaluations were performed to determine the bacterial load. After Gram staining, the slides were screened (magnification 1000×) and the bacterial count was reported. The semi-quantitative reporting system was subdivided from + (<1) to +++ (>100) counts per ocular field. The microbiological test results were categorized as “infected”, or “non-infected”, by looking for the presence of potential pathogenic microorganisms (ppmos) relative to the general microbiological flora.

### 3.2. Functionalization of Fast Blue RR

Fast blue RR (N-(4-amino-2,5-dimethoxyphenyl) benzamide) was coupled to alkoxysilanes for immobilization. The coupling was performed according to Hasmann [8] and the molecular structure of the synthesized substrate was proven by 1H and 13C nuclear magnetic resonance (NMR). NMR spectra were measured on a Bruker Avance 3 (Bruker, Bremen, Germany) at 300 MHz for protons and 75 MHz for 13C. The derivatized Fast Blue RR was dissolved in DMSO-d6. Sixteen scans were accumulated for 1H and 2048 scans for 13C.

### 3.3. Immobilization of Derivatized Fast Blue RR

As a model surface for hydrolytic polymerization of alkoxysilane-derivatized Fast Blue RR silica thin-layer chromatography (TLC) plates were used. 1 cm × 1 cm squares were cut and overlaid with a 50 mM solution of derivatized Fast Blue RR dissolved in EtOH. The polymerization was performed for 24 h at 105 °C.

### 3.4. Transformation of the Substrate

The transformation of alkoxysilane-derivatized Fast Blue RR was carried out using pure myeloperoxidase (MPO, planta), infected and non-infected wound fluid (wf) of ulcer wounds and hemoglobin (Sigma, St. Louis, MO, USA). Wound fluids were collected as described in Schiffer *et al*. [6]. MPO was diluted in potassium phosphate buffer (100 mM; pH 7) to (end) concentrations of 20 U/mL; 10 U/mL; 5 U/mL. The wound fluids were diluted in potassium phosphate buffer (100 mM; pH 7) or sodium acetate buffer (100 mM; pH 4), and hemoglobin was used in end concentrations of 0.01 mM, 0.05 mM and 0.001 mM. The wound fluids and the hemoglobin dilutions were additionally measured at 405 nm to compare the hemoglobin content of the wound fluid and the hemoglobin solutions.

Ten µl of the respective solutions were mixed with ten µl of 39.2 mM H_2_O_2_ solution and pipetted on the silica plates with the immobilized substrate. Solutions lacking H_2_O_2_ were used as blanks.

Additionally, the visual inspection of the silica plates after 30 min and 60 min, the color changes on the surface of the silica plates were measured with a ColorLite sph850 spectrophotometer (ColorLite GmbH, Katlenburg-Lindau, Germany). As a reference, a wetted silica plate with immobilized substrate, but without enzyme was used. Based on the delta E values, calculations and statistical analyses were performed. To compare the different experimental setups regarding the statistical significance, Two-sample t-tests assuming equal variances were performed. A p-value of less than 0.001 was considered as statistical significant.

### 3.5. HPLC Sample Treatment

The sample preparation of HPLC analysis was carried out as follows. The working solutions (1 mL) contained 20 mM of the derivatized Fast Blue RR, 1.5 U/ mL MPO, 39.2 mM H_2_O_2_, 50 mM NaCl in 100 mM potassium phosphate buffer (pH 7) or sodium acetate buffer (pH 4). As negative controls, all samples were also prepared leaving out MPO. The samples were incubated for 0, 15, 30, 45, 60, 90, 180, and 360 minutes. After these time points, 1 mL absolute EtOH was added and the acidic samples were adjusted to a pH of 7. A Carrez clarification was performed. 20 µL of C1 solution (5.325 g of K_4_[Fe(CN)_6_]*3 H_2_O, dissolved in water filled up to a volume of 50 mL) was added to the samples and vortexed for 1 minute. Subsequently 20 µL of C2 solution was added (14.400 g of ZnSO_4_*7 H_2_O, dissolved in water, filled up to a volume of 50 mL) 5 min shaked and an centrifuged (15,000 rpm) for 15 min. Five hundred µL of the samples were again mixed with 500 µL of EtOH absolute and purified using a 45 µm filter. The samples were then distributed (250 µL each) in HPLC vials.

### 3.6. HPLC Measurement

#### 3.6.1. LC

A LC 1260 pump (Agilent G1312B, Palo Alto, CA, USA) was operated using 20 mM ammonia formiate in water and acetonitrile as mobile phase. A gradient was set from 0% to 100% acetonitrile within 35 min in a 45 min method. The Column Poroshell 120 EC-C18 4.6 mm × 50 mm 2.7 Micron (Agilent, Palo Alto, CA, USA) was equilibrated at 40 °C in a 1290 Infinity 2 TCC (Agilent G7116B) with 80% water 20 mM ammonia formiate and acetonitrile for 60 min. The LC-MS grade water was purified by an ELGA PURELAB ultra (VWS). All other chemicals were supplied from Sigma Aldrich (St. Louis, MO, USA) in LC-MS grade.

#### 3.6.2. LC-ESI TOF

The LC was coupled to a DAD (Agilent G4212B, Palo Alto, CA, USA) and a Dual ESI G6230B TOF (Agilent, Palo Alto, CA, USA). For the electrospray ionization (operating in positive ion mode), a nebulizer was used, the dry gas flow was set to 8 L/min and a pressure of 40 psig at 250 °C was chosen. The fragmenter voltage was set to 200 V, the skimmer at 65 V, the octopole to a voltage of 750 V and the reference masses were 121.0509 m/z and 922.0098 m/z. Ions from 50 m/z to 3000 m/z were acquired with the Agilent MassHunter Workstation (Version B06.01, Palo Alto, CA, USA). The DAD signal at 254 nm was also monitored by the Agilent MassHunter Workstation. A statistical calculation in accordance to German industrial standard 32645 for the detection limit, detectability limit, and limit of determination was performed. Significance was tested with p-values less than 0.05.

## 4. Conclusions

Fast Blue RR acts as a suitable marker for the detection of infection. Upon incubation with MPO or infected wound fluid, a blue color development was observed on polymerized alkoxysilane-derivatized Fast Blue RR layers. Cross reactions with hemoglobin occur but delta E values as well as the visual inspection confirm a negligible color development compared to pure MPO solutions or infected wound fluids. LC-ESI TOF analyses further confirm the conversion of the substrate by MPO at different pH values. This derivatized substrate could facilitate the determination of infection in wounds.
